# Causal relationship of excess body weight on cardiovascular events through risk factors

**DOI:** 10.1038/s41598-022-08812-x

**Published:** 2022-03-28

**Authors:** Thosaphol Limpijankit, Prin Vathesatogkit, Dujrudee Matchariyakul, Sirichai Wiriyatanakorn, Sukanya Siriyotha, Ammarin Thakkinstian, Piyamitr Sritara

**Affiliations:** 1 Division of Cardiology, Department of Medicine, Faculty of Medicine Ramathibodi Hospital, Mahidol University, 270 Rama VI Road, Ratchathewi, Bangkok, 10400 Thailand; 2 Medical and Health Office, Electricity Generating Authority of Thailand, Bangkruay, Nonthaburi, 11130 Thailand; 3 Section for Clinical Epidemiology and Biostatistics, Faculty of Medicine Ramathibodi Hospital, Mahidol University, Bangkok, Thailand

**Keywords:** Cardiology, Health care, Risk factors

## Abstract

Excess body weight is associated with cardiovascular events (CVEs) and premature death. This study aimed to find the causal pathways between excess body weight and CVEs through risk factors in a general adult population. A total of 7921 employees of the Electricity Generating Authority of Thailand were enrolled during 1997–2009. Baseline characteristics and blood test results were collected. A body mass index (BMI) ≥ 23 kg/m^2^, using WHO criteria for Asians was defined as excess body weight. A mediation analysis was applied to assess potential causal pathways. BMI ≥ 23 kg/m^2^ was considered as an independent variable, whereas diabetes mellitus (DM), hypertension (HT), and chronic kidney disease (CKD) were considered as mediators, and CVEs (i.e., fatal and non-fatal coronary artery disease or stroke) were considered as the outcomes. The prevalence of BMI ≥ 23 kg/m^2^, DM, HT, and CKD were 62.7%, 7.8%, 28.1% and 11.8% respectively. During an average of 17.2 ± 5.5 years follow-up, subjects with BMI ≥ 23 kg/m^2^ compared with those with lower BMIs more frequently developed CVEs (9.4 vs 6.2%, *P* < 0.001). The effects of BMI ≥ 23 kg/m^2^ on CVEs were mediated indirectly through DM and HT with significant ORs of 1.61 (1.34, 2.09) and 1.57 (1.39, 1.80), respectively. The indirect effect of CKD on CVEs was significantly increased if mediated through DM → HT or HT [ORs of 1.17 (1.09, 1.32) and 1.20 (1.10, 1.32), respectively]. Subjects with excess body weight were prone to develop CVEs which were mediated indirectly through DM and HT. The effect of CKD on CVEs was small but enhanced if it occurred as a complication of DM or HT.

## Introduction

Overweight and obesity, hereafter called ‘excess body weight’, are major public health problems worldwide, and associated with cardiovascular events (CVEs) and premature death^1,2^. Despite increased awareness, this epidemic problem continues growing with the prevalence of overweight and obesity in adults (aged 18 years or older) reaching 39% and 13%, respectively, as per the WHO report in 2016^3^. People with excess body weight are prone to develop type 2 diabetes mellitus (T2DM), dyslipidemia, hypertension (HT) and so-called ‘metabolic syndrome’ and to experience premature atherosclerotic cardiovascular disease (CVD) and death^4,5^.

Dyslipidemia is associated with atherosclerotic CVD. The linkage between dyslipidemia and atherogenesis is supported by a strong correlation between low-density lipoprotein cholesterol (LDL-C) and major adverse CVEs^6^. Reduction in LDL-C improves CVD outcomes whether achieved through primary or secondary prevention^7,8^. While a causal link between dyslipidemia and CVD is well established, such a causal association between excess body weight and CVD remains controversial. This is important because the increasing prevalence of excess body weight may eventually offset the public health gains achieved by improved treatment of CVD.

Much evidence show that elevated body mass index (BMI) increases the risk of T2DM and insulin resistance^9,10^. In obese subjects, increased amount of non-esterified fatty acids, glycerol, proinflammatory substances, and hormones are involved in the development of insulin resistance and thus T2DM development^11^. Obesity-related HT is another important pathway to increased CVEs, and previous evidence shows a strong association of BMI with HT^12,13^. As the prevalence of excess body weight increases, the prevalence of HT with its associated CV risk increases as well. The pathophysiology of obesity-related HT is insulin stimulation of the sympathetic nervous system with increased renin release, increased angiotensinogen release from intra-abdominal adipocytes and increased aldosterone production, leading to increased sodium reabsorption and obstructive sleep apnea^14^.

In addition to T2DM and HT, there is a well-known relationship between BMI and deterioration renal function^15^. Numerous population-based studies have shown an association between obesity and progression of chronic kidney disease (CKD)^16–18^. The possible mechanisms include excessive excretory loads, lipid accumulation in the kidney, metabolic abnormalities in the adipose tissue, and over activation of the renin–angiotensin–aldosterone system^19^. However, the exact mechanisms through which excess body weight may worsen or cause CKD remain unclear. Certainly, there is a significantly higher prevalence of T2DM and HT among excess body weight patients with CKD (both early and late stage). Some of the worsening renal function in obesity may be mediated by downstream comorbid conditions such as T2DM or HT.

As for complex mechanisms among these diseases, there is a hypothesis that excess body weight is associated with CVEs, and that it is probably mediated through T2DM, HT or CKD. No study to date has demonstrated a causal relationship between T2DM, HT or CKD in excess body weight subjects and CVEs. Mediation analysis offers a tool to assess the magnitude of different pathways leading to an outcome^20^. This study was conducted in a general adult population using mediation analysis to explore the possibility of a causal relationship between excess body weight and CVEs; if found, whether it was mediated directly or indirectly through T2DM, HT, or CKD.

## Methods

### Study design

The Electricity Generating Authority of Thailand (EGAT) study is a large prospective cohort study conducted in adult employees to better understand the occurrence and influence of risk factors on CVD occurrence and progression in adult Thais. This longitudinal health study is composed of three cohorts (EGAT 1, 2, and 3) with follow-up every five years; the cohort profiles are published^21^. Briefly, employees were enrolled in the Bangkok metropolitan areas (EGAT 1 and 3) and at three different sites in Western and Northern Thailand (EGAT 2). The age at enrollment ranged from 35 to 54 years, to maximize the probability of a CVD event, given that the retirement age in EGAT was 55 years. A large variety of people were recruited, from illiterates working as cleaners to truck drivers, security guards, office clerks, administrators, architectures, engineers, field explorers, lecturers, lawyers, health-care practitioners and executive board members. Inclusion criteria allowed for inclusion of asymptomatic employees with risk factors for atherosclerosis (but no CVD symptoms). Exclusion criteria included: (1) a diagnosis CVD at baseline [either coronary artery disease (CAD) or stroke], and (2) no baseline BMI measurement.

The study protocol conformed to the ethical guidelines of the 1975 Declaration of Helsinki and was approved by the Ethics Committee of the Faculty of Medicine, Ramathibodi Hospital, Mahidol University (# COA. No. MURA2019/402). All participants provided their written informed consent. This cohort study was reported following the STROBE guidelines^22^.

### Subjects and data collection

A total of 7921 subjects from the EGAT 1, 2, and 3 cohorts were enrolled in 1997, 1998, and 2009, respectively. Baseline characteristics, including age, sex, atherosclerosis risk factors (e.g., T2DM, HT, dyslipidemia, smoking, and alcohol), BMI (kg/m^2^) and underlying diseases, were recorded. To characterize metabolic risk factors, fasting blood glucose (FPG), lipid profile [total cholesterol, high-density lipoprotein cholesterol (HDL-C), LDL-C, triglyceride (TG)] and uric acid, were collected from these subjects. These baseline characteristic data were used for further analyses.

### Study factor of interest

BMI was the study factor of interest, measured at baseline, was calculated as weight in kilograms divided by height in meters squared (kg/m^2^). It was categorized according to the WHO criteria for Asians^23^ as underweight (< 18.5 kg/m^2^), normal weight (18.5–22.9 kg/m^2^), overweight (23–24.9 kg/m^2^), or obese (≥ 25 kg/m^2^); these two later groups were then combined as ‘excess body weight’ and used to compare with the other former groups (‘non-excess body weight’) for further analysis.

### Mediators and clinical outcomes

Mediators of interest were T2DM, HT, and CKD, and were measured both at baseline and during follow up, but only baseline-enrolment data were used for the analysis. T2DM was defined as either an overnight FPG ≥ 126 mg/dL or taking anti-diabetic medications^24^. HT was defined as either systolic BP (SBP) ≥ 140 mmHg and/or diastolic BP (DBP) ≥ 90 mmHg, or taking anti-hypertensive medication^25^. An estimated GFR (mL/min/1.73m^2^) was calculated based on the CKD Epidemiology Collaboration (CKD-EPI) equations for men and women^26^. CKD was defined as an eGFR < 60 mL/min/1.73 m^2^.

The primary endpoints were CVEs (i.e., fatal or non-fatal CAD, fatal or non-fatal stroke, and all-cause death (ACD)^27^. CAD was defined as the presence of at least one of the following criteria: angina, acute coronary syndrome, acute myocardial infarction (MI), a significant (> 70% diameter stenosis) coronary lesion on angiography, revascularization [i.e., percutaneous coronary intervention (PCI) or coronary artery bypass graft (CABG)], and documented myocardial ischemia during exercise testing. Stroke was defined as a history of ischemic or hemorrhagic stroke, or transient ischemic attack (TIA). If a subject had two CVEs, only the first event was used for analysis. If a subject died of a CVE, this event was counted as both a death and a CVE. These outcomes were assessed at baseline and during follow up (i.e., years 2017, 2018, and 2019 for EGAT1, EGAT2, and EGAT3, respectively) but only new occurrences were used in the overall analysis. Participants who lost to follow-up were linked with death databases [maintained by the National Health Security Office (which contained hospital discharge records) and the Department of Provincial Administration of the Ministry of the Interior (which contained death certificates)] to ascertain their vital status. Each cause of death was assessed by an independent adjudication committee, comprised of cardiologists and neurologists, and classified into CAD (fatal CAD or MI or sudden unexplained death), stroke (including ischemic, hemorrhagic and subarachnoid hemorrhage), other CVD death [e.g. heart failure, valvular heart disease or peripheral arterial disease (PAD)], non-CVD death [i.e., malignancy, respiratory and gastrointestinal diseases, accident, sepsis, metabolic (T2DM, HT, DLP)], or unknown.

### Data collection

The data were initially collected and recorded in case record forms (CRFs). Then, they were computerized by well-trained research assistants. All electronic databases were stored at a central data management unit, Department of Clinical Epidemiology and Biostatistics, Faculty of Medicine, Ramathibodi Hospital. Data cleaning and checking were regularly performed after each survey. In addition, cross-linked conditions were developed to ensure the accuracy and consistency of the data.

### Statistical analysis

The characteristics of patients were expressed as means [± standard deviation (SD)] or median (range) (when SD was half of the mean or greater) for continuous variables and percentages for categorical variables, respectively. A univariate analysis was performed to assess the relationship of each variable with CVD using t–tests and Chi-square tests for continuous and categorical variables, respectively.

**Figure 1:**
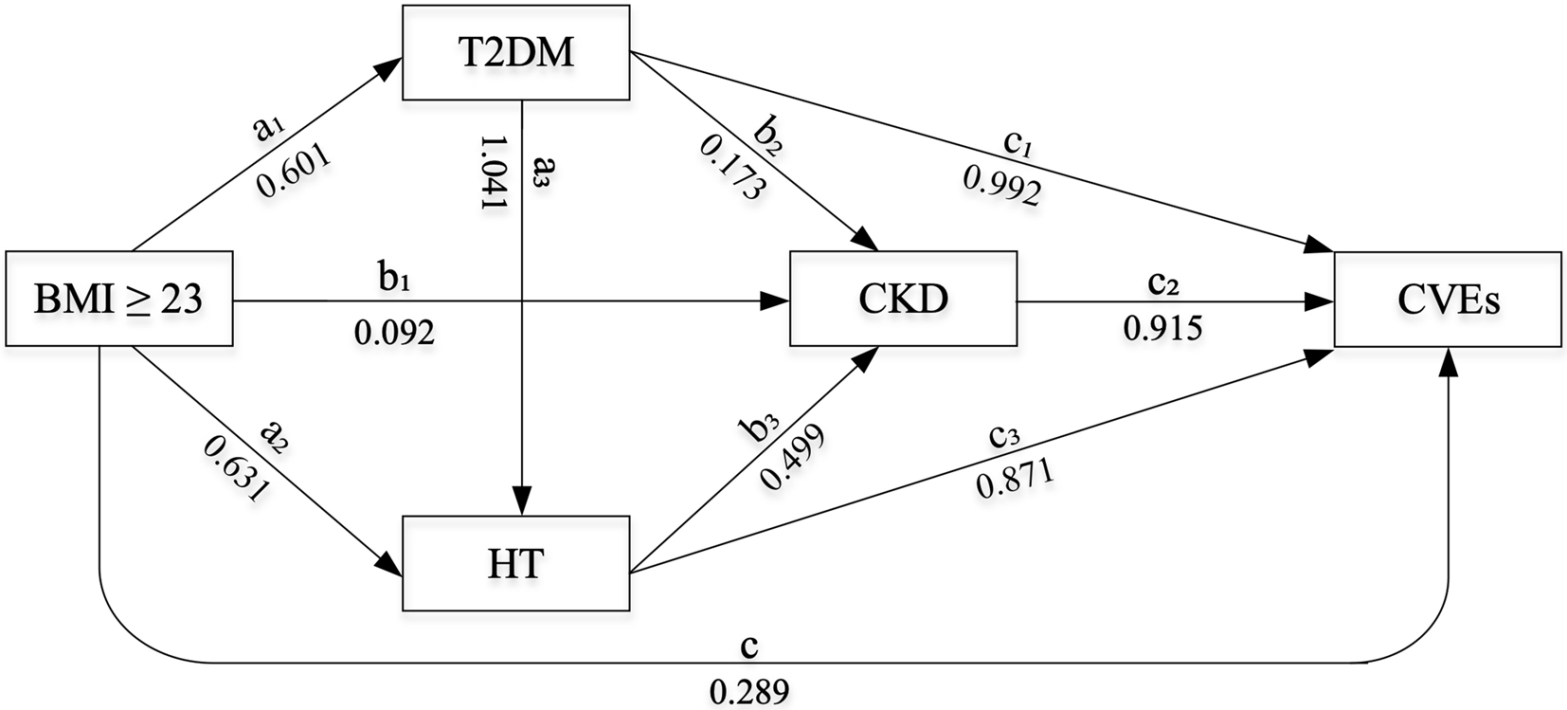
Causal pathway diagrams of excess body weight and CVEs through T2DM, HT, and CKD mediators.

Mediation analysis using a generalized structural equation model (GSEM) was applied to assess potential causal pathways as illustrated in Fig. 1. Excess body weight (BMI ≥ 23 kg/m^2^) was considered as the independent variable, whereas the binary variables T2DM, HT, and CKD were considered as mediators, and CVEs were considered as the outcome of interest. Three mediation models and one outcome model were constructed as follows^28–30^:

Mediation models

path a1 $$ {\text{ln}}\left[ {\frac{{P\left( {T2DM} \right)}}{{1 - P\left( {T2DM} \right)}}} \right] = a_{0} + a_{1} Excess \,body\, weight + \mathop \sum \nolimits_{k} e_{k} z_{k} $$

path a2 $$ {\text{ln}}\left[ {\frac{{P\left( {HT} \right)}}{{1 - P\left( {HT} \right)}}} \right] = a_{0} + a_{2} Excess \,body \,weight\, + \,a_{3} TDM + \mathop \sum \nolimits_{k} e_{k} z_{k} $$

path b1b2b3 $$ {\text{ln}}\left[ {\frac{{P\left( {CKD} \right)}}{{1 - P\left( {CKD} \right)}}} \right] = a_{0} + b_{1} Excess\, body\, weight + b_{2} T2DM + b_{3} HT + \mathop \sum \nolimits_{k} e_{k} z_{k} $$

Outcome model:

$$ \begin{aligned} {\text{ln}} \left[ {\frac{{P\left( {CVEs} \right)}}{{1 - P\left( {CVEs} \right)}}} \right] = a_{0} + cExcess\, body \,weight + c_{1} DM + c_{2} HT + c_{3} CKD  +  \\ \mathop \sum \nolimits_{k} e_{k} z_{k} ; \,where \,z_{k} = confounders \end{aligned}$$

P(T2DM), P(HT), P(CKD), and P(CVEs) are probability of occurrence of T2DM, HT, CKD, and CVEs, respectively. The GSEM with logit link function was used to regress T2DM and HT on excess body weight (called paths a_1_ and a_2_). Then, the CKD mediation model was constructed to regress CKD on excess body weight, T2DM, and HT using logit link (called path b_1_, b_2_, and b_3_). Next, excess body weight and the three mediators were fitted on the CVEs outcome using a logit link (path c, c_1_, c_2_, and c_3_). Finally, three mediation and outcome models were simultaneously constructed using the GSEM approach. Confounders whose p-values were less than 0.10 in the univariate analysis of each mediation and outcome models were simultaneously considered, but only significant confounders were finally retained in the multivariate GSEMs.

Potential causal mediation effects were then estimated using the products of coefficients by the final mediation and outcome GSEM models^31^; the odds ratios (ORs) of mediation effects were then estimated^32^. A bootstrap method with 5000 replications was applied to estimate mediation effects along with 95% confidence intervals (CIs) based on a bias-corrected technique^33^. All analyses were performed using STATA version 16. A *P*-value of less than 0.05 was considered to be statistically significant.

## Results

A total of 7921 out of the 8028 participants had baseline BMI and were then used for analysis. Distribution of baseline characteristics of participants grouped by BMI (i.e., BMI < 23 kg/m^2^ and ≥ 23 kg/m^2^) are summarized in Table 1. Participants with excess body weight were more frequently male, T2DM, HT, CKD, ex/current smokers, ex/current drinkers and had higher levels of LDL-C, triglyceride and uric acid, but lower HDL-C. During an average of 17.2 ± 5.5 years of follow-up, excess body weight participants developed CVEs (via either CAD or stroke) more frequently than did those with non-excess body weight (Table 2). However, frequency of ACD, death from cancer and CAD were not significantly different between the two groups.

**Table 1:** Characteristics of the study population at baseline, grouped as with and without excess body weight.

Baseline characteristics	Excess body weight (BMI ≥ 23)		*P*-value
	Yes (n = 4969)	No (n = 2952)	
**Age, n (%)**			
Age ≥ 60	323 (6.5)	175 (5.9)	0.310
Age < 60	4646 (93.5)	2777 (94.1)	
**Gender, n (%)**			
Male	3825 (77.0)	2102 (71.2)	< 0.001
Female	1144 (23.0)	850 (28.8)	
**T2DM, n (%)**			
Yes	459 (9.2)	156 (5.3)	< 0.001
No	4506 (90.8)	2793 (94.7)	
**HT, n (%)**			
Yes	1633 (32.9)	595 (20.2)	< 0.001
No	3335 (67.1)	2356 (79.8)	
**CKD, n (%)**			
Yes	616 (12.6)	316 (10.8)	0.021
No	4271 (87.4)	2597 (89.2)	
**Smoking status, n (%)**			
Ex or current smoke	2360 (47.5)	1281 (43.4)	< 0.001
Never smoke	2607 (52.5)	1669 (56.6)	
**Alcohol status, n (%)**			
Ex or current drinker	2745 (55.4)	1506 (51.1)	< 0.001
Never drinker	2214 (44.6)	1441 (48.9)	
LDL-C, mg/dl, mean (SD)	155.1 (39.4)	146.5 (38.2)	< 0.001
HDL-C, mg/dl, mean (SD)	50.2 (11.5)	56.3 (13.7)	< 0.001
Triglyceride, mg/dl, median (range)	138.0 (15.0, 1587.0)	102.0 (15.9, 2076.0)	< 0.001
Uric acid, mg/dl, mean (SD)	6.0 (1.5)	5.4 (1.5)	< 0.001

Mediators	Excess body weight (BMI ≥ 23)		*P*-value
	Yes (n = 4969)	No (n = 2952)	
**T2DM, n (%)**			
Yes	459 (9.2)	156 (5.3)	< 0.001
No	4506 (90.8)	2793 (94.7)	
**HT, n (%)**			
Yes	1633 (32.9)	595 (20.2)	< 0.001
No	3335 (67.1)	2356 (79.8)	
**CKD, n (%)**			
Yes	616 (12.6)	316 (10.8)	0.021
No	4271 (87.4)	2597 (89.2)	

*BMI* body mass index, *CKD* chronic kidney disease, *CVEs* cardiovascular events, *HDL-C* high-density lipoprotein cholesterol, *HT* hypertension, *LDL-C* low-density lipoprotein cholesterol, *T2DM* diabetes mellitus.

T-test was used for comparing means, Mann–Whitney for medians, Chi-square test for categorical data.

**Table 2:** Long-term clinical outcomes evaluated at the end of follow-up.

Clinical outcomes	Excess body weight (BMI ≥ 23)		*P*-value
	Yes (n = 4969)	No (n = 2952)	
**CVEs, n (%)**			
Yes	465 (9.4)	183 (6.2)	< 0.001
No	4504 (90.6)	2769 (93.8)	
**CAD, n (%)**			
Yes	289 (5.8)	115 (3.9)	< 0.001
No	4680 (94.2)	2837 (96.1)	
**Stroke, n (%)**			
Yes	194 (3.9)	80 (2.7)	0.005
No	4775 (96.1)	2872 (97.3)	
**ACD, n (%)**			
Yes	707 (14.2)	398 (13.5)	0.354
No	4262 (85.8)	2554 (86.5)	
**Cancer death, n (%)**			
Yes	217 (30.7)	132 (33.2)	0.396
No	490 (69.3)	266 (66.8)	
**Vital status, n (%)**			
Alive	4262 (85.8)	2554 (86.5)	0.167
CAD death	207 (4.2)	98 (3.3)	
Non-CAD death	500 (10.1)	300 (10.2)	

*ACD* all-cause death, *CAD* coronary artery disease, *CVEs* cardiovascular events.

### Relationships between risk factors and BMI

To find factors associated with T2DM, HT, CKD and CVEs, univariate regression analyses were performed (see Table 3). BMI ≥ 23 kg/m^2^, older age and ex/current smoking were common and associated with T2DM, HT, CKD and CVEs. For other factors, there was inconsistency of association. Male gender, HDL-cholesterol and triglyceride were each associated with T2DM, HT and CVEs, but not with CKD. Ex or current drinking was associated with T2DM, HT, CKD, but not with CVEs. LDL-cholesterol was associated with T2DM and CKD, but not with HT nor CVEs. Uric acid was associated with HT, CKD and CVEs, but not with T2DM.

**Table 3:** Factors associated with diabetes mellitus, hypertension, chronic kidney disease and cardiovascular events: univariate logistic regression.

Factors	T2DM		OR	95% CI	*P*-value
	Yes (n = 615)	No (n = 7299)			
BMI ≥ 23	459 (9.2)	4506 (90.8)	1.824	1.512, 2.200	< 0.001
Age ≥ 60	66 (13.3)	431 (86.7)	1.916	1.458, 2.518	< 0.001
Male	518 (8.8)	5402 (91.3)	1.875	1.500, 2.344	< 0.001
Ex or current smoke	356 (9.7)	3281 (90.2)	1.682	1.424, 1.986	< 0.001
Ex or current drinker	377 (8.9)	3869 (91.1)	1.417	1.197, 1.679	< 0.001
LDL-C, mg/dl, mean (SD)	146.7 (43.7)	152.4 (38.8)	0.996	0.994, 0.998	0.001
HD-C, mg/dl, mean (SD)	48.9 (11.9)	52.7 (12.7)	0.973	0.966, 0.980	< 0.001
Triglyceride, mg/dl, median (range)	160.1 (43.4, 933.0)	121.2 (15.0, 2076.0)	1.003	1.002, 1.004	< 0.001
Uric acid, mg/dl, mean (SD)	5.9 (1.6)	5.8 (1.5)	1.050	0.993, 1.111	0.086

Factors	HT		OR	95% CI	*P*-value
	Yes (n = 2288)	No (n = 5691)			
BMI ≥ 23	1633 (32.9)	3335 (67.1)	1.939	1.741, 2.159	< 0.001
T2DM, n (%)	317 (51.5)	298 (48.5)	3.007	2.545, 3.551	< 0.001
Age ≥ 60	205 (41.2)	293 (58.8)	1.867	1.551, 2.248	< 0.001
Male	1889 (31.9)	4037 (68.1)	2.283	2.007, 2.597	< 0.001
Ex or current smoke	1163 (31.9)	2478 (68.1)	1.418	1.285, 1.564	< 0.001
Ex or current drinker	1357 (31.9)	2893 (68.1)	1.508	1.365, 1.666	< 0.001
LDL-C, mg/dl, mean (SD)	151.2 (40.4)	152.2 (38.7)	0.999	0.998, 1.001	0.345
HDL-C, mg/dl, mean (SD)	50.7 (12.5)	53.2 (12.7)	0.984	0.980, 0.988	< 0.001
Triglyceride, mg/dl, median (range)	147.8 (23.9, 1273.0)	116.0 (15.0, 2076.0)	1.003	1.002, 1.004	< 0.001
Uric acid, mg/dl, mean (SD)	6.3 (1.5)	5.6 (1.5)	1.370	1.323, 1.419	< 0.001

Factors	CKD		OR	95% CI	*P*-value
	Yes (n = 932)	No (n = 6868)			
BMI ≥ 23	616 (12.6)	4271 (87.4)	1.185	1.026, 1.369	0.021
T2DM, n (%)	92 (15.3)	508 (84.7)	1.370	1.085, 1.730	0.008
HT, n (%)	355 (16.3)	1829 (83.7)	1.694	1.469, 1.954	< 0.001
Age ≥ 60	176 (36.5)	306 (63.5)	4.992	4.087, 6.098	< 0.001
Male	714 (12.3)	5107 (87.7)	1.129	0.961, 1.327	0.139
Ex or current smoke	463 (13.1)	3080 (86.9)	1.216	1.061, 1.395	0.005
Ex or current drinker	419 (10.1)	3726 (89.9)	0.689	0.600, 0.790	< 0.001
LDL-C, mg/dl, mean (SD)	156.8 (40.4)	151.2 (39.0)	1.004	1.002, 1.005	< 0.001
HDL-C, mg/dl, mean (SD)	52.5 (11.5)	52.5 (12.9)	1.000	0.995, 1.006	0.937
Triglyceride, mg/dl, median (range)	131.0 (31.0, 1587.0)	122.0 (15.0, 2076.0)	1.000	0.999, 1.001	0.369
Uric acid, mg/dl, mean (SD)	6.5 (1.6)	5.7 (1.5)	1.354	1.292, 1.419	< 0.001

Factors	CVEs		OR	95% CI	* P*-value
	Yes (n = 648)	No (n = 7273)			
BMI ≥ 23, kg/m^2^	465 (9.4)	4504 (90.6)	1.562	1.308, 1.866	< 0.001
T2DM, n (%)	128 (20.8)	487 (79.2)	3.426	2.766, 4.245	< 0.001
HT, n (%)	331 (14.9)	1897 (85.1)	2.958	2.514, 3.481	< 0.001
CKD, n (%)	465 (6.8)	6403 (93.2)	2.768	2.275, 3.368	< 0.001
Age ≥ 60, n (%)	114 (22.9)	384 (77.1)	3.830	3.053, 4.805	< 0.001
Male n (%)	568 (9.6)	5359 (90.4)	2.536	1.995, 3.223	< 0.001
Ex or current smoke, n (%)	387 (10.6)	3254 (89.4)	1.830	1.553, 2.155	< 0.001
Ex or current drinker, n (%)	354 (8.3)	3897 (91.7)	1.050	0.893, 1.235	0.554
LDL-C, mg/dl, mean (SD)	153.2 (39.2)	151.8 (39.2)	1.001	0.999, 1.003	0.384
HDL-C, mg/dl, mean (SD)	49.8 (10.6)	52.7 (12.8)	0.981	0.974, 0.988	< 0.001
Triglyceride, mg/dl, median (range)	144.0 (38.1, 1587.0)	122.0 (15.0, 2076.0)	1.002	1.001, 1.003	< 0.001
Uric acid, mg/dl, mean (SD)	6.3 (1.6)	5.8 (1.5)	1.267	1.200, 1.338	< 0.001

Abbreviations as in Table 1.

Multivariate GSEMs were constructed simultaneously, considering BMI ≥ 23 kg/m^2^ along with these covariates (see Table 4). After adjusting covariables, BMI ≥ 23 kg/m^2^ was significantly associated with T2DM, HT and CVEs but not CKD, with the corresponding estimated ORs (95% CI) of 1.68 (1.39, 2.04), 1.76 (1.57, 1.97), and 1.34 (1.10, 1.62), 1.09 (0.938, 1.269), respectively. This could be interpreted as excess body weight participants being 1.68, 1.76, and 1.34 times more likely to develop T2DM, HT, and CVEs than subjects with non-excess body weight.

**Table 4:** Factors associated with mediators and cardiovascular events outcome: multivariate GSEMs.

Model	Factors	OR	SE	Z-test	*P*-value	95% CI
T2DM	BMI ≥ 23	1.685	0.165	5.33	< 0.001	1.391, 2.041
	Age ≥ 60	1.811	0.258	4.17	< 0.001	1.370, 2.395
	Male	1.343	0.169	2.33	0.020	1.048, 1.719
	Ex or current smoke	1.342	0.127	3.10	0.002	1.114, 1.616
HT	BMI ≥ 23	1.759	0.101	9.86	< 0.001	1.572, 1.968
	T2DM	2.484	0.219	10.34	< 0.001	2.090, 2.951
	Age ≥ 60	1.718	0.171	5.45	< 0.001	1.414, 2.088
	Male	1.813	0.132	8.15	< 0.001	1.571, 2.092
	Ex or current drinker	1.154	0.066	2.53	0.011	1.033, 1.290
	Triglyceride	1.002	0.0002	8.18	< 0.001	1.001, 1.002
CKD	BMI ≥ 23	1.091	0.084	1.13	0.257	0.938, 1.269
	T2DM	1.106	0.140	0.79	0.427	0.863, 1.418
	HT	1.588	0.122	6.00	< 0.001	1.365, 1.847
	Age ≥ 60	4.558	0.479	14.42	< 0.001	3.709, 5.601
	Ex or current smoke	1.343	0.106	3.74	< 0.001	1.151, 1.568
	Ex or current drinker	0.589	0.047	-6.69	< 0.001	0.505, 0.688
	LDL-C	1.004	0.001	3.94	< 0.001	1.002, 1.005
CVEs	BMI ≥ 23	1.336	0.132	2.94	0.003	1.101, 1.621
	T2DM	2.512	0.296	7.83	< 0.001	1.995, 3.164
	HT	2.217	0.198	8.91	< 0.001	1.860, 2.641
	CKD	2.010	0.219	6.41	< 0.001	1.624, 2.488
	Age ≥ 60	2.787	0.354	8.06	< 0.001	2.172, 3.576
	Male	1.895	0.273	4.43	< 0.001	1.428, 2.514
	Ex or current smoke	1.424	0.140	3.59	< 0.001	1.174, 1.728
	Ex or current drinker	0.678	0.065	− 4.05	< 0.001	0.562, 0.818
	HDL-C	0.992	0.004	− 2.08	0.038	0.984, 1.000

Abbreviations as in Table 1.

*CI* confident interval, *OR* odd ratio, *SE* standard deviation.

In addition, participants with T2DM were 2.48 (2.09, 2.95) and 2.5 (1.99, 3.16) times more likely to develop HT and CVEs, respectively, whereas the risk for developing CKD was not significant [OR of 1.106 (0.863, 1.418)]. Participants with HT were 1.6 (1.36, 1.84) and 2.2 (1.86, 2.64) times more likely to develop CKD and CVEs, respectively. Participants with CKD had a significant association with having a CVE [OR of 2.01 (1.62, 2.49)]. Other factors associated with an increased risk of CVEs were age ≥ 60, male gender, and being an ex/current smoker. As noted, high HDL and ex/current alcohol drinking were protective factors for CVEs.

### Mediation analysis

Indirect effects of BMI ≥ 23 kg/m^2^ on CVEs through T2DM, HT and CKD were further estimated using a bootstrapping analysis with 5000 replications, see Table 5. The three mediation pathways that yielded the highest ORs were BMI ≥ 23 kg/m^2^ → T2DM, BMI ≥ 23 kg/m^2^ → HT, and BMI ≥ 23 kg/m^2^ → T2DM → HT with ORs of 1.62 (1.34, 2.09), 1.57 (1.39, 1.80), and 1.46 (1.25, 1.78), respectively. This could be interpreted as excess body weight participants being about 1.34 (1.11, 1.67) times more likely to develop CVEs, and their risk being increased to 1.62, 1.57 and 1.46 if they had T2DM, HT, and T2DM plus HT, respectively. Interestingly, the effects of BMI ≥ 23 kg/m^2^ on CVEs that were mediated through CKD and T2DM → CKD were insignificant, with ORs of 1.063 (0.962, 1.195) and 1.037 (0.956, 1.149), respectively. However, the effect of excess body weight on CVEs through HT and CKD did reach significance if mediated through HT (BMI ≥ 23 kg/m^2^ → HT → CKD) or T2DM (BMI ≥ 23 kg/m^2^  → DM → HT → CKD), which yielded ORs of 1.20 (1.10, 1.32) and 1.16 (1.08, 1.32), respectively.

**Table 5:** Estimation of mediation effects and contribution of each pathway relative to total effects of excess body weight on cardiovascular events.

Paths	Effect	95% CI	Percentage of effect contributed	95% CI
BMI ≥ 23 → T2DM → CVEs	1.617	1.337, 2.092	23.7	16.4, 31.6
BMI ≥ 23 → T2DM → HT → CVEs	1.459	1.252, 1.779	18.6	12.4, 24.4
BMI ≥ 23 → T2DM → CKD → CVEs	1.037	0.956, 1.149	1.8	0.1, 5.6
BMI ≥ 23 → T2DM → HT → CKD → CVEs	1.166	1.086, 1.325	7.5	4.7, 12.3
BMI ≥ 23 → HT → CVEs	1.568	1.393, 1.798	22.1	15.9, 29.8
BMI ≥ 23 → HT → CKD → CVEs	1.200	1.104, 1.321	9.0	5.1, 14.1
BMI ≥ 23 → CKD → CVEs	1.063	0.962, 1.195	3.0	0.2, 8.2
BMI ≥ 23 → CVEs	1.336	1.111, 1.667	14.3	5.7, 24.3

*BMI* body mass index, *CKD* chronic kidney disease, *CVEs* cardiovascular events, *T2DM* diabetes mellitus, *HT* hypertension.

Effects of excess body weight on CVEs contributed through T2DM, HT, and T2DM plus HT were 29.1%, 27.2%, and 18.6%, respectively. In addition, the effects of excess body weight contributed through CKD alone was 3.0%. However, effects contributed through HT → CKD or DM → HT → CKD were 9.0% or 7.5%, respectively. As noted, the direct effect of excess body weight on CVEs was found to be 14.3% of the contribution effect.

## Discussion

This study was designed to explore the potential causal relationship between excess body weight and CVEs, using known risk factors (including T2DM, HT and CKD) as mediators based on a 17-year longitudinal cohort. The results from mediation analysis suggested that excess body weight itself increased the risk of CVEs about 34%, and increased further to 57%, 62% and 46% if mediated through HT, T2DM, and T2DM plus HT, respectively.

A WHO panel reported that Asian populations are at risk for T2DM and CVD at lower BMIs than the standard WHO criteria of a BMI exceeding 25^23^. The American Diabetes Association recently recommended that testing for T2DM should be considered for all Asian American adults with a BMI of ≥ 23^34^. This cut-off is consistent with a previous report from Japan that a BMI ≥ 23 is a risk factor for insulin resistance and T2DM^10^. Based on these sources, a BMI ≥ 23 was used as a definition of excess body weight in this cohort.

The prevalence of overweight or obesity was quite significant, found in nearly two-thirds of participants. The majority of these excess body weight participants were middle-aged males; they more frequently had atherosclerotic risk factors (T2DM, HT, CKD, ex/current smoking or alcohol use, dyslipidemia and hyperuricemia). This cluster of risk factors, the so-called ‘metabolic syndrome’, are positively associated with incidence of CVEs and all-cause deaths^35^. During the long-term follow-up, the excess body weight subjects developed CVEs (both non-fatal CAD and non-fatal strokes) more frequently than other subjects. These findings are similar to the previous reports of higher CVEs in overweight and obesity populations^2,36^. Unlike reports in previous literatures^37^, there was no significant increase in CAD and cancer deaths in this excess body weight population. This difference raises questions that will require further follow-up of the cohort to resolve.

### Relationship between excess body weight and CVEs through mediators

The clinical prognosis of participants with excess body weight ranges from mild to severe. In fact, some overweight and obesity people never develop a CVE nor die prematurely. As many as 25% of obese individuals are classified as “metabolically healthy”, suggesting that increased body weight alone is not sufficient to cause adverse events^38^. Such individuals usually are mildly obese, young and without complications. Our aim was to find causal relationships between excess body weight and CVEs, whether mediated directly or indirectly through the most likely clinical risk factors.

Besides dyslipidemia and smoking which have well-established correlations with CVEs, three other major risk factors that potentially have causal links between excess body weight and CVEs are T2DM, HT and CKD. After adjusting for confounders, this cohort study found that these three risk factors still mediated an increase in the effect of excess body weight on CVE risk. As mentioned previously, excess body weight results in complex metabolic abnormalities and may cause a high incidence of T2DM, HT and CKD. If these chronic conditions are not modified, CVEs and premature death can occur.

Behavior, environment, and genetic factors all have roles in causing people to be overweight and obese. Duration and severity of excess body weight, and concurrent control measures, all influence the occurrence of complications. A majority of patients are unsuccessful at changing their life-style and continue their unhealthy habits. This study showed that the strongest mediators associated with CVEs are T2DM and HT. Subjects with excess body weight were more likely than those without to develop T2DM, HT, or T2DM plus HT, resulting in increased development of CVEs by 1.62, 1.57, and 1.46 times, respectively. CKD was not a strong mediator causing CVEs in contrast to T2DM and HT. It is possible that our study population included patients with early-stage CKD (eGFR < 60 mL/min/1.73 m^2^) which had less impact on CVE occurrence. However, if CKD occurs as a complication in patients who have already developed T2DM plus HT, or HT alone, it increases the risk of CVEs.

### Direct effect of excess body weight on CVEs

As noted, the direct effect of excess body weight itself on CVEs is about 1.34 times more than subjects with under- or normal body weight, without mediation through T2DM, HT or CKD. This unidentified mechanism might be explained by genetics, inflammatory processes, hyperuricemia or decreased HDL-C level. There is evidence that accumulation of visceral fat mass increases the production of pro-inflammatory cytokines and adipokines, and can promote the formation of atherosclerotic plaques^39,40^. Moreover, obesity tends to increase the risk for CVEs by increasing macrophage infiltration and instability of plaques^41,42^. Hyperuricemia is another confounding factor that is reported to predict the development of atherosclerosis. It is frequently found in obese subjects and carries a high risk of subclinical CVD^43,44^. Patients with metabolic syndrome usually have hyperuricemia along with other clinical abnormalities. Elevated serum uric acid has been shown to be a predictor of coronary calcium deposition independent of conventional CV risk factors^45^. This finding suggests that serum uric acid might be part of mechanisms which increase the risk of CVEs in excess body weight populations.

In addition, obesity is an important risk factor for decreased HDL-C levels, which predisposes to CVD^46^. There is evidence that intra-abdominal visceral fat deposition is an important negative correlate of HDL-C level^47^. HDL reduces coronary atherosclerosis by decreasing the expression of adhesion molecules on endothelial cells and thereby reducing inflammation, and by inhibiting the oxidation of LDL-C. The HDL-C level is somewhat difficult to increase, since it is mostly determined by genetics. In current practice, decreasing of LDL-C with statins and exercise is the only way to counterbalance this abnormality.

### Clinical implication

The implication of this mediation analysis in terms of prevention is to encourage excess body weight patients to reduce their BMI to prevent early onset T2DM and HT, and also possibly to slow renal function deterioration and increase life expectancy. Early detection of all conventional risk factors and then modifying behavior and changing life-style are appropriate ways to reduce CVEs. These measures decrease disease progression and likelihood of premature death. The term of ‘metabolically healthy’ overweight and obesity seems to not be absolutely correct, because in this study subjects with BMI ≥ 23 kg/m^2^ also carry some risk of CVEs, even in the absence of T2DM, HT and CKD.

### Strength and limitation

The strengths of this study include its prospective nature, large population and long duration of follow-up. Also, all clinical events were reviewed and verified by an expert committee. The EGAT project is still continuing. Its follow-up surveys every five years will continue to document clinical outcomes and thus enable the answering of some open questions. Also, this analysis included all confounding factors which might impact on CVEs and was adjusted for these factors as much as possible.

Several limitations of the study need to be mentioned. First, the association study between excess body weight and CVEs relied on the BMI and covariables at a single time point which makes it difficult to adequately address bias associated with reverse causality. Second, the study population was enrolled only from among EGAT employees who were mostly middle aged with higher socioeconomic status and education than the general population. Our findings thus may not be generalizable to those of younger age nor to the whole population. In addition, this study did not examine whether concurrent treatment or intervention with life-style modifications during the follow-up had any effect on the occurrence of CVEs. Lastly, the BMI that was used to group participants was determined at the first day of survey. Participant’s BMI may have evolved during the follow-up period.

## Conclusions

Excess body weight is a major health problem in Thailand and likely a cause of CVE occurrences. Participants who were overweight or obese trended to develop T2DM and HT leading to occurrence of CVEs during long-term follow-up. Although effects of excess body weight through CKD seems to have been minimal, it may be enhanced if it occurred as a complication of DM and HT. It is important that healthy overweight and obese people should reduce their BMIs to prevent T2DM and HT, and so increase likelihood of remaining free of disease and preventing premature death. Concurrent reduction in the magnitude of all conventional risk factors is the key for successfully reducing the occurrence of CVEs.

## Data Availability

The data that support the findings of this study are available from the corresponding author upon reasonable request.
